# Relationship Dysfunction in Couples When One Partner Is Diagnosed with Borderline Personality Disorder: Findings from a Pilot Study

**DOI:** 10.3390/bs13030253

**Published:** 2023-03-13

**Authors:** Julia Kroener, Caroline Schaitz, Alexander Karabatsiakis, Anna Maier, Bernhard Connemann, Elisa Schmied, Zrinka Sosic-Vasic

**Affiliations:** 1 Christophsbad Goeppingen, Research Division of Applied Psychotherapy and Psychiatry, Faurndauer Straße 6-28, 73035 Goeppingen, Germany; 2 Department of Psychiatry and Psychotherapy III, University Clinic of Ulm, 89075 Ulm, Germany; 3 MSB Medical School Berlin, Psychotherapeutic Outpatient Facility, Rüdesheimer Straße 50, 14197 Berlin, Germany; 4 Department of Psychology, Clinical Psychology II, University of Innsbruck, 6020 Innsbruck, Austria

**Keywords:** borderline personality disorder, BPD, relationship dysfunction, HPA, HPG, testosterone, cortisol, stress, childhood trauma

## Abstract

Relationship dysfunction—marked by frequent conflicts—is one of the hallmark features of borderline personality disorder (BPD). However, the BPD couple as a dyad and partner-related features have rarely been taken into account. The aim of the present study was to investigate hormonal, personality, and relationship relevant factors, such as relationship satisfaction, attachment, and trauma in both partners within a dyad where one partner is diagnosed with BPD. The total sample consisted of 26 heterosexual couples. All studies were conducted at 2 p.m. Primary outcomes: Neo-Five-Factor-Inventory, Childhood Trauma Questionnaire, Experiences in Close Relationships Scale. Secondary outcomes: Problem List, Partnership Questionnaire, Questionnaire for Assessing Dyadic Coping. Upon questionnaire completion, one saliva sample was taken via passive drool to assess baseline cortisol and testosterone levels. Results showed that females with BPD have higher scores on childhood maltreatment, dysfunctional attachment styles, and neuroticism than mentally healthy females. Furthermore, they have more relationship-related problems and are less satisfied in their romantic relationship. Male partners of women with BPD showed lower testosterone levels, higher levels of childhood maltreatment, dysfunctional attachment styles, neuroticism, and openness compared with the healthy control partners. Furthermore, childhood trauma, neuroticism as well as dysfunctional attachment styles displayed a significant positive correlation with relationship-related problems. Traumatic childhood experiences, insecure attachment styles as well as neurotic personality characteristics contribute to increased relationship disruptions in couples. Relevant hormonal and psychosocial parameters in BPD partners should be taken into account when treating females with BPD.

## 1. Introduction

With a prevalence of 2.7% within the general population [1], and a prevalence of 10% within psychiatric outpatients as well as up to 22% in psychiatric inpatients [2], borderline personality disorder (BPD) is amongst the most prevalent and cost-intensive mental disorders within public healthcare systems [3,4]. BPD is characterized by various, heterogeneous symptoms, such as emotion dysregulation, impulsivity, fear of abandonment, and hostility, which can contribute to a multitude of relationship dysfunctions [5]—one of the hallmark features of the disorder [6,7]. However, whilst there are several studies evaluating the above-described symptoms [8,9,10,11], relatively little research has been conducted around relationship dysfunctions in BPD in general. This fact is rather surprising considering that past research demonstrated the effects of interpersonal conflicts or difficulties on BPD symptoms (e.g., emotion dysregulation, impulsivity, or hostility), ultimately leading to emotional crises (e.g., suicide attempts) within this population and the subsequent need for professional care [12,13]. 

Thus far, there have been three predominant theories attempting to explain interpersonal dysfunctions in BPD: (1) Attachment theories postulate that traumatic childhood experiences (i.e., experiences of abuse and neglect) with primary caregivers are at the core of the development of insecure attachment styles, which remain comparably stable throughout life [14,15,16,17]. This theory is closely aligned with findings from BPD studies that reveal elevated rates of childhood maltreatment [18,19,20], as well as high levels of insecure attachment styles [21,22,23] within the disorder, possibly contributing to relationship dysfunctions [23]. (2) Drawing and extending on the previous theory, the third wave of cognitive behavioural therapy (e.g., schema therapy) postulates an association between dysfunctional experiences with primary caregivers during childhood and early adolescence and the development of negative relationship schemes during adulthood, such as isolation or distrust, which can act as inner working models, recreating dysfunctional relationship experiences [24,25]. (3) Lastly, biological models underlying relationship dysfunction in BPD assume a possible dysregulation of the autonomic nervous system (ANS) and the hypothalamic–pituitary–adrenal (HPA) axis, resulting from chronic stress exposure during childhood and adolescence, which can lead to difficulties in coping with relationship distress on a cognitive, emotional, as well as behavioural level [26,27,28,29]. This theory is supported by current research revealing abnormal patterns of HPA and ANS activation in patients with BPD in response to stress [30], which in turn affect adaptive behavioural and emotional coping strategies during times of heightened stress exposure [31,32]. However, while these three theories are seemingly distinct, they are rather connected: Traumatic as well as severely dysfunctional experiences with primary caregivers are at the core of each of the above-postulated theories, resulting in biological dysregulations of the stress response system and the subsequent development of insecure attachment styles as well as maladaptive inner schemes. 

Nevertheless, there are still some major gaps in current research on relationship dysfunctions in BPD: First, past findings on romantic relationships in BPD-free CG couples have shown that several distinct clinical variables, such as emotional intimacy, communication patterns, or the degree of commitment [33,34] in *both* partners contribute to a healthy relationship. Therefore, it is surprising that research on BPD has mainly focused on dysfunctional variables in the person suffering from BPD, rather than considering both partners within the dyad and their shared interactions together, despite preliminary evidence suggesting that the BPD partner might contribute to symptom intensity as well as treatment prognosis in BPD patients [23,35,36]. Supporting this finding, Bouchard and colleagues [37] revealed that 55.9% of partners living with individuals diagnosed with PBD also fulfilled the diagnostic criteria for at least one personality disorder, which in turn has been linked to symptom maintenance in the partner diagnosed with BPD [38]. Furthermore, several studies demonstrated that up to 95% of BPD partners display insecure (anxious) attachment styles [37,39,40], suggesting the presence of dysfunctional experiences with primary caregivers not only in individuals with BPD but also in their partners. 

Taking these findings together, it might not be surprising that research has found an increase in domestic violence in heterosexual couples, with the female partners suffering from BPD reporting higher rates of domestic abuse than women without BPD [37,41,42], as well as higher levels of re-victimization by their romantic partner [41,42]. Interestingly, at the same time, the majority of women with BPD also reported being physically or emotionally violent towards their romantic partners [37], with BPD symptoms being linked to the experience of physical abuse in BPD partners [12,42]. However, while domestic abuse might be tied to personality and attachment characteristics within BPD couples, to date, there has been no research investigating the hormonal underpinnings of externalizing behaviour, hostility, impulsivity, and aggression within BPD relationships. This is somewhat curious since past research has associated these features with elevated levels of testosterone, the end product of the hypothalamic–pituitary–gonadal (HPG) axis [43,44,45]. This axis in turn is connected to the stress response system (HPA-axis; [46]), with the HPA and ANS axes being known to show several dysregulations in patients with BPD, such as a blunted stress response towards socio-evaluative threat [26,30,47]. Aligning with this notion, previous research on testosterone and BPD revealed elevated baseline testosterone levels in women with BPD [48,49], a possible result of a dysregulated androgen metabolism within this sample [46]. Furthermore, HPG-axis dysregulation has been associated with traumatic childhood experiences as well as chronic exposure to stress during developmentally relevant stages [50], with both factors commonly experienced by individuals with BPD [20,51,52,53]. Lastly, testosterone plays a crucial role in conjunction with dominance and submission (e.g., [54]), both of which have been shown to be relevant within the context of relationship dysfunction in BPD [55].

Drawing on previous scientific findings and gaps in research on relationship dysregulation in BPD, it is apparent that several factors need to be taken into account to gain a more holistic picture of relationship dysfunction and possible underlying factors in BPD. Therefore, the current study aimed to investigate possible underpinnings of couple dysfunction in BPD from various angles: (1) Females with BPD as well as their romantic male partners were examined; (2) several traits of each partner within the couple were taken into account (e.g., traumatic childhood experiences); and (3) hormonal underpinnings (i.e., testosterone and cortisol levels) were investigated. Based on previous research, in our primary hypothesis, we assume that both females with BPD as well as their male partners experience increased levels of insecure attachment styles, higher CM load, as well as higher levels of neurotic personality traits compared with CG females and males, respectively. Furthermore, due to the high amount of shared aggression within BPD couples, in our second hypothesis we postulate that testosterone levels are elevated in BPD patients as well as their partners compared with BPD-free female and male controls. Aligning with previous scientific findings, in our second hypothesis, we additionally expected to find lower cortisol levels in BPD patients compared with female controls. Our third hypothesis proposes that females and males in the BPD couple group display lower levels of couple satisfaction, as well as healthy dyadic coping styles, and more relationship-related problems compared with mentally healthy females and their partners. Lastly, our fourth hypothesis suggests that relationship-related problems are positively correlated with the predispositioning factors described in our primary hypothesis. 

## 2. Materials and Methods

### 2.1. Participants

We recruited 26 heterosexual couples, amongst whom, two couples had to be excluded from endocrinological analysis due to impure saliva samples (e.g., severe discoloration of the sample, contamination with blood). Therefore, the final dataset for the endocrinological analysis consisted of N = 24 couples: n = 12 couples where the female participant was diagnosed with BPD, and n = 12 BPD-free control couples where the female participant did not report any current or past mental health diagnosis (see Table 1 for cohort descriptives and characteristics). For the analysis of self-reported measurements, the four previously excluded participants were included, leading to N = 26 couples (n = 14 couples where the female participant was diagnosed with BPD, and n = 12 BPD-free control couples). 

Female BPD patients who are currently in a romantic relationship were recruited within the in- and outpatient facilities of the Department of Psychiatry and Psychotherapy III at the University Clinic of Ulm, whereas the healthy couples were recruited through public announcements (e.g., at the University of Ulm, on social media). 

Diagnostic criteria for females with BPD were assessed using the Structured Clinical Interview for DSM-IV Axis II [56], whereas Axis-I comorbidities were evaluated using the Mini-International Neuropsychiatric Interview (M.I.N.I.) [57]. Additionally, mood dysregulations, as well as general health parameters, were assessed with the Beck Depression Inventory (BDI-II) [58] as well as the General Health Questionnaire (GHQ-12) [59], respectively, to further characterize the study participants. Male partners did not receive a standardized diagnostic interview (i.e., M.I.N.I.); however, they completed the same questionnaires for sample characterization as their female counterparts (i.e., BDI-II, GHQ-12, SCID-II). The female participants recruited for the control group were excluded if they met diagnostic criteria for any Axis-I disorder or BPD—assessed by the same clinical interviewers as for the female BPD patients—or reported any current or previous psychiatric or psychotherapeutic treatment, as well as current intake of any psychiatric medication. Females on birth control were included in the study with this variable being later statistically controlled, while females who were not taking any birth control were assessed during the follicular phase of their menstrual cycle to allow for comparability of hormonal parameters. Both couple groups (BPD and BPD-free controls) were required to be aged between 18–55 years, be in a heterosexual romantic relationship for at least 6 months, and were excluded if they reported any regular past or current substance or medication abuse or dependency within the past three months. In addition, any somatic or autoimmune diseases, endocrine disorders, or current infections were used as exclusion criteria. In addition, currently pregnant or breastfeeding were used as exclusion criteria. Females with BPD were required—if they were prescribed psychiatric medications—to have medication intake stabilized for at least three months. Furthermore, females suffering from BPD were excluded if they had any comorbid diagnosis of eating disorders, psychotic disorder, or bipolar disorder. 

The study was approved by the Ethics Committee of the Medical Department of the University of Ulm and in accordance with the latest version of the Declaration of Helsinki. All participants provided written informed consent before study participation. Female BPD patients and participants of the control group as well as their male partners received monetary compensation (EUR 35 and EUR 20, respectively) for study participation. 

### 2.2. Procedure

After an initial phone screening, female participants (i.e., HC and BPD) were invited to a diagnostic session, where the M.I.N.I and SCID-II interviews were conducted by a trained clinical psychologist one week before the laboratory appointment with their partner. Each of the laboratory visits was scheduled at 2 p.m. to account for diurnal hormonal variations according to recommendations in the literature [60,61]. Upon arriving at the laboratory, all participants provided written informed consent and measurements of their digits were taken to investigate the 2D:4D ratio [62]. Afterwards, participants were asked to complete a standardized test battery. To assess possible pre-experimental stress effects due to traveling to the appointment [63,64], one baseline saliva sample was taken 45 min after arriving at the laboratory. All female participants either took hormonal contraceptives on the day of the laboratory visit or were scheduled during the luteal phase of their menstrual cycle to account for hormonal cycle variations. Furthermore, before the laboratory visit, all participants were asked to adhere to certain requirements to minimize possible cofounds influencing hormonal parameters (e.g., refrain from heavy physical exercise, coffee, alcohol, or pro re nata medication intake 24 h before the laboratory visit).

### 2.3. Measurements

#### 2.3.1. Primary Outcomes

Experiences in Close Relationships-Revised (ECR-RD) [65]: The ECR is a self-report questionnaire assessing attachment styles by asking the participants about how they experience romantic relationships in general based on 36 items, rated on a 7-point Likert scale, ranging from 1 (disagree completely) to 7 (agree completely). The items are divided into two subsets of 18 items each, measuring two dimensions of the attachment system, namely attachment avoidance (e.g., “I don’t feel comfortable opening up to my romantic partners”) and attachment anxiety (e.g., “I often worry that my partner does not want to stay with me”). The ECR displays good psychometric properties, with an internal consistency of Cronbach’s α = 0.92 for the overall scale, and a Cronbach's α = 0.81 to 0.89 for the two subscales [65]. 

Childhood Trauma Questionnaire—Short Form (CTQ-SF) [66]: The CTQ-SF is a self-report measurement assessing adverse experiences during childhood (childhood maltreatment, CM) with 31 items, which are rated on a 5-point Likert scale ranging from 1 (never true) to 5 (very often true). To capture the overall experience of childhood maltreatment (CM load), a sum score can be built across all items (except for the control items in the subscale trivialization). Additionally, six subscales can be built to assess specific types of CM, such as emotional and physical neglect as well as emotional, physical and sexual abuse, and the experience of inconsistency. The CTQ-SF has good psychometric properties, with internal consistencies ranging from Cronbach's α = 0.85 to 0.94 for the overall score, and Cronbach´s α = 0.70 to 0.93 for the respective subscales [67,68]. 

Neo-Five Factor Inventory (NEO-FFI) [69]: The NEO-FFI is a 60-item measurement assessing the big-five personality traits extraversion, openness to experiences, conscientiousness, neuroticism, and agreeableness, which are considered to be common personality traits across the world and therefore independent from cultural or linguistic representations. The self-report measurement is presented on a 5-point Likert scale ranging from 1 (strongly disagree) to 5 (strongly agree). There is no overall sum score; however, each of the five personality domains is assessed at the hand of 12 distinct items. Internal consistencies across the subscales range from Cronbach´s α = 0.71 to 0.85 [69].

#### 2.3.2. Secondary Outcomes

Problem List (PL-S, German Original: [70]; English Version: [71]). The PL-S is a 23-item self-report measurement evaluating possible areas of conflict within a couple´s relationship (e.g., “Habits of one's partner”, “Sexuality”, or “Household chores”). Participants are instructed to indicate on a 4-point Likert-scale if there is a conflict in the evaluated area, and if yes, if there is a successful resolution (score 1), or recurring conflicts around the topic (score 2), or if there is conflict, but the couple does not talk about it (score 3). The PL displays good psychometric properties, with an internal consistency of Cronbach´s α = 0.82 for the overall scale. 

Partnership Questionnaire (German: Partnerschaftsfragebogen, PFB; [72]). The PFB is a 30-item self-report measurement assessing the quality of couple´s relationships. Each item is rated on a 4-point Likert-scale ranging from 0 (never) to 3 (very often). To assess overall relationship satisfaction, a sum score can be built across all items. Additionally, three subscales, with 10 items each, can be built to assess display of affection, sense of togetherness, and conflict behavior. The PFB has good psychometric properties with Cronbach's α ranging from 0.88 to 0.92 [72]. 

Questionnaire for Assessing Dyadic Coping (QADC-2R) [73]. The QADC-2R is a 48-item self-report measure assessing the couple´s attempt to cope with common everyday marital stressors (e.g., “We share everyday chores, so we both have similar daily workloads”). Each item can be answered on a 4-point Likert-scale ranging from 0 (never) to 3 (most of the time). Within the questionnaire, an overall score assessing shared dyadic coping efforts can be built. Additionally, two subscales evaluating positive as well as negative dyadic coping strategies (e.g., signaling of one´s own emotional distress, hostile dyadic coping) can be integrated. The QADC has excellent psychometric properties with Cronbach´s α ranging from 0.86 to 0.93.

### 2.4. Sampling, Processing, and Analysis of Saliva Samples

Each couple provided their baseline saliva sample approximately at 2.45 p.m. (M = 2.46; SD = 0.02), due to the diurnal rhythm of hormonal parameters. In order to avoid dilution or contamination of saliva, participants were asked to refrain from drinking 10 min prior to providing the saliva sample. Additionally, participants were asked to refrain from drinking alcohol or caffeine, and from participating in any strenuous physical activity 24 h prior to the laboratory visit. Moreover, participants were instructed to refrain from eating (including gum, lollipops etc.), smoking, and to solely consume natural drinking water two hours prior to entering the laboratory. Saliva samples were collected via passive drool using Salivettes (Salimetrics, LLC) and stored immediately at −20 °C, until they were sent frozen and in batches to the laboratory of Clemens Kirschbaum at the University of Dresden for laboratory analysis every six months. The saliva samples were assayed for testosterone and cortisol in singlets, with 20% of samples measured in duplicates, to test the reliability of the analysis, using a commercially available enzyme immunoassay kit (Salimetrics, LLC). The coefficient of variation (CV) for all assayed hormones was ≤5% for intra-assay variance. 

### 2.5. Data Analysis

Analyses investigating between-group differences were conducted with SPSS (Version 28.0), implementing mixed modeling using maximum likelihood (ML) estimation with Satterthwaite approximation. For each of the outcome measures, a linear mixed model with the condition as the between group factor, as well as a random intercept for subject, was implemented. As the random effect was comprised of one level, the variance components (VC) covariance structure was specified to model the covariance structure of the intercept. No imputation of missing values was used as the mixed model approach assumes that values are missing at random, therefore providing unbiased parameter estimates. Within each model, the effect of group was entered. Between group differences were tested using pairwise contrasts. Furthermore, standard deviations, confidence intervals (CIs), and effect sizes (Cohen´s d) were extracted out of the mixed model analysis based on marginal means. The comparison between groups was provided by the estimate for the fixed effect (displayed as F-statistic) from the mixed model. For the general as well as between couples correlational analysis (BPD couples vs. HC couples), Pearson’s correlation coefficient was implemented. 

### 2.6. Sample Characteristics

Table 1 displays results for the sample characteristics. The female groups (BPD and controls) did not differ regarding age, BMI, level of school education, the intake of oral contraceptives, or current smoking status. Females with BPD reported significantly higher scores of depressive symptoms (BDI-II), more health issues (GHQ-12), as well as more Personality Disorders (SCID-II-Questionnaire) than women of the control group. Within the BPD group, n = 8 women received a single psychiatric medication, and n = 4 patients reported no current psychiatric medication intake. Additionally, there was a significant difference between the two groups regarding overall relationship duration.

The partners of the females with BPD and the partners of the BPD-free women did not differ regarding age, BMI, level of education, smoking status, general health parameters, or number of personality disorders (Table 2). Amongst the partners of the BPD females, n = 1 partner took a single psychiatric medication, and n = 11 partners of females with BPD did not take any psychiatric medications. All male partners in the control group were free of any psychiatric medication.

## 3. Results

### 3.1. Predispositioning Factors

Comparing the female participants (BPD vs. HC), there was a main effect for group for attachment anxiety (ECR) (*F*(26,1) = 72.14, *p* < 0.001), as well as attachment avoidance (*F*(26,1) = 13.81, *p* < 0.001) (Table 3; Supplementary Materials). Additionally, there was a main effect for group regarding the total amount of CM load (CTQ) (*F*(1,26) = 24.46, *p* < 0.001), with BPD females displaying significantly higher levels of childhood maltreatment than their healthy counterparts (Table 3; Supplementary Materials). Moreover, females with BPD experienced significantly higher scores on each of the CTQ subscales (all *F* > 9, all *p* < 0.01), except for a trend finding for physical neglect (for details see Table 3 and Supplementary Materials). Lastly, there was a main effect for group between BPD and CG females on various personality domains (NEO-FFI; Table 3; Supplementary Materials). Specifically, there was a main effect for group for neuroticism, with BPD females displaying significantly higher scores than their healthy female counterparts. Furthermore, there was a main effect for group for extraversion, conscientiousness, as well as agreeableness between BPD and CG females, with BPD females demonstrating significantly lower scores than their female counterparts. BPD and CG females did not differ regarding the personality trait openness (*F* < 3, *p* > 0.05).

Investigating differences between BPD and CG partners, there was a main effect for group regarding attachment anxiety (ECR) (*F*(21,1) = 3.97, *p* ≤ 0.05), as well as attachment avoidance (*F*(21,1) = 5.54, *p* < 0.05), with partners of BPD females displaying significantly higher scores on both scales (Table 3; Supplementary Materials). Additionally, there was a main effect for group comparing male BPD and CG partners for CM load (CTQ) (*F*(21,1) = 7.15, *p* ≤ 0.01), with BPD partners displaying significantly higher values for overall CM load than CG partners. All the results on childhood maltreatment and the associated subscales are reported in Table 3. Furthermore, there was a main effect for group regarding the personality traits neuroticism and openness, with the BPD partner group displaying significantly higher scores than the CG partners (Table 3; Supplementary Materials). Moreover, BPD partners displayed lower scores on the scale for extraversion compared with CG partners on a trend level, while the scales for conscientiousness and agreeableness remained non-significant.

### 3.2. Hormone Levels in Saliva

There was no main effect for group regarding testosterone levels between the female groups, indicating that females with BPD (*M* = 24.81; *SD* = 13.06) did not significantly differ from females of the control group (*M* = 28.60; *SD* = 16.34), (*F*(24,1) = 0.47, *p* = 0.50). Additionally, there was no main effect for group regarding cortisol levels between females with BPD (*M* = 3.70; *SD* = 1.87) and females in the control group (*M* = 4.76; *SD* = 3.46), (*F*(24,1) = 1.06, *p* = 0.31). 

**Figure 1:**
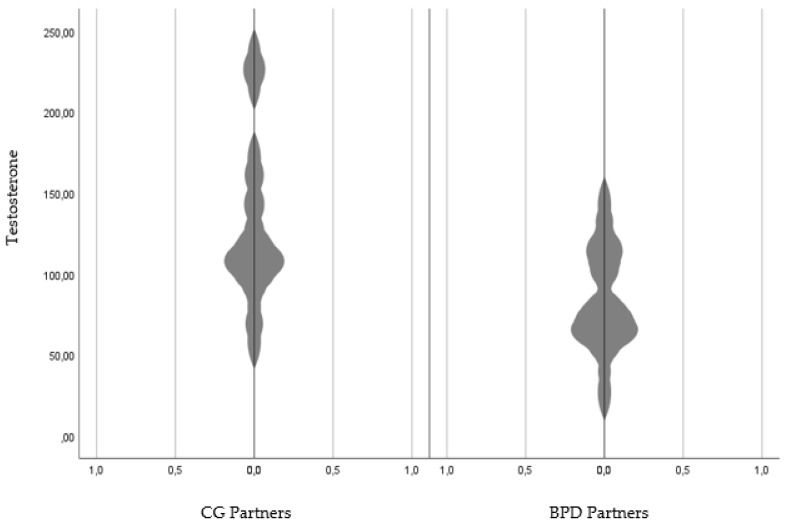
Testosterone levels in BPD partners compared with CG partners. *Note:* CG = Control group; BPD = Borderline personality disorder.

Among the male partners, there was a significant difference between the partners of the BPD females and the partners of the control group females regarding their testosterone levels, with the male partners of the control group showing significantly higher testosterone levels than the BPD partners (*F*(22,1) = 8.18), *p* < 0.01) (Figure 1). There was no main effect for group comparing BPD partners (M = 3.88; SD = 2.61) and CG partners (M = 4.83; SD = 2.59) regarding their cortisol levels (*F*(22,1) = 0.87, *p* = 0.36). 

### 3.3. Relationship Parameters

Results are presented in Table 4. Looking at overall relationship satisfaction (PFB) among females with and without a BPD diagnosis, there was a main effect for group (*F*(26,1) = 5.45, *p* < 0.05), indicating lower scores of relationship satisfaction in the female BPD group. Additionally, there was a significant main effect for overall dyadic coping (FDCT), and negative dyadic coping, as well as for positive dyadic coping on a trend level, revealing lower levels of overall and positive dyadic coping in females with BPD, as well as higher levels of negative dyadic coping. Lastly, there was a main effect for group for overall relationship problems (PL Sum), with more relationship problems in females with BPD compared with CG females. 

Comparing the male partners of both groups, no main effects for group were found, an all the results remained non-significant (all *F* < 1.5, all *p* > 0.05) (Table 4).

When looking at relationship parameters in the BPD as well as the HC couples, no significant differences were observed within the BPD couples (all *F* < 3, all *p* > 0.05) (for means and standard deviations, see Table 4). Within the HC couples, female CG participants had significantly higher values on the scales for relationship satisfaction (*F*(24,1) = 8.04, *p* < 0.01) (PFB Sum) and for overall dyadic coping (*F*(24, 1) = 6.22, *p* < 0.05) (FDCT sum), as well as significantly lower values on relationship-related problems (*F*(24,1) = 5.15, *p* < 0.05) (PL Sum) and negative dyadic coping (*F*(24,1) = 6.77, *p* < 0.05) (FDCT negative coping; see Table 4 for means and standard deviations). No significant differences were found within the CG couples for positive coping (*F*(24,1) = 2.88, *p* = 0.10) (FDCT positive coping).

The correlational analysis can be found in Table 5. There was no statistical correlation between relationship problems and attachment styles or CM load for females diagnosed with BPD. However, for their male partners, attachment anxiety, sexual abuse, as well as overall CM load displayed a significant positive correlation with relationship-related problems. Within the healthy control group, females showed a strong positive correlation between relationship problems and attachment avoidance, emotional abuse, physical abuse, emotional neglect, as well as physical neglect. Lastly, the partners of the female CG displayed a significant positive correlation between relationship problems and attachment avoidance as well as physical neglect.

## 4. Discussion

The aim of the present study was to investigate relationship dysfunction in BPD from various angles. First, we evaluated predispositioning factors, which could possibly contribute to relationship dysfunction, such as personality traits, CM load and attachment styles. We assumed that both partners within the BPD dyad would experience higher prevalence rates of insecure attachment styles and CM load as well as more dysfunctional personality traits compared with couples within the healthy control group. Second, we assessed possible predispositioning endocrinological parameters leading to relationship dysfunction. Our second hypothesis states that testosterone levels would be elevated, whereas cortisol levels would be blunted in BPD patients and their partners compared to females and males in the control group. Third, we examined relationship relevant parameters, such as relationship-related problems, relationship satisfaction, and dyadic coping, assuming that females and males within the BPD dyad would display lower levels of relationship satisfaction and functional coping styles, as well as more relationship-related problems than their female and male counterparts within the healthy control group. Lastly, we assumed that relationship problems would be associated with the predispositioning factors described in our second assumption. 

Looking at the subjective reports of both groups as assessed by self-reports (i.e., attachment, personality, and childhood maltreatment), we were able to confirm our hypothesis: BPD females, as well as their male partners, experienced higher levels of attachment avoidance and attachment anxiety than their respective male and female counterparts in the control group. This finding aligns with previous research [7,22,23,37], indicating that BPD is associated with higher prevalence rates of insecure attachment. The experience of inconsistent, emotionally withdrawn, unavailable, or abusive relationships is a possible underlying cause for the development of insecure attachment styles [74,75], which in turn, result in an inner working model automatically guiding possible expectations of future relationships. These inner working models mirroring insecurity and uncertainty of relationships with significant others ultimately contribute to one of the core symptoms of BPD: anxiety of abandonment as well as frantic efforts to avoid the possibility thereof. However, based on findings in this study, male romantic partners of females with BPD similarly experience high levels of insecure attachment styles, indicating that it might not only be the female diagnosed with BPD who is holding dysfunctional core beliefs, which could contribute to problems within romantic relationships. In the present study, we could furthermore confirm an association between relationship problems and insecure attachment styles in general, which further supports the idea that relationship problems are related to insecure attachment styles. This result also aligns with previous scientific findings showing high rates of anxious/insecure attachment styles in BPD partners ranging between 31.4 and 95% [26,76]. However, to our knowledge, there are no scientific findings evaluating the prevalence of insecure attachment styles in BPD partners at the beginning, as well as after a certain period of the relationship, with a partner suffering from BPD. Therefore, it remains unclear whether partners of women with BPD were experiencing insecure attachment styles before entering the relationship with a person diagnosed with BPD, or whether these feelings of insecurity are fostered and possibly increased by their relationship experience. While attachment styles are found to remain comparably stable throughout life, they might be altered or intensified by positive as well as negative relationship experiences [77,78,79], and therefore, more research is warranted within the context of relationships where one partner is diagnosed with BPD. This thought is linked to another finding by Allison and colleagues [76] who assumed that the high levels of anxious attachment in the BPD partner was present to serve a certain purpose: It resembles the partner´s desire to prevent extreme behavioral reactions in the partner with BPD, such as anger outbursts, self-mutilation, or threats of suicide. 

Linked to the above-presented findings, females with BPD as well as their male partners reported higher overall CM loads compared with their female and male counterparts in the control group. Specifically, females with BPD experienced more CM on all presented subscales than females in the control group with large effect sizes. This result is supported by a recent meta-analysis [80] revealing that BPD patients are 13 times more likely to report a history of CM compared to healthy participants. Specifically, 48.9% of patients with BPD stated that they experienced physical neglect, while 42.5% reported emotional abuse, 36.4% physical abuse, 32.1% sexual abuse, and 25.3% emotional neglect [80]. This notion aligns with the above-presented theory around BPD assuming an association between CM and the development of insecure attachment styles, with both factors contributing to relationship dysfunction in BPD [81]. This theory was further confirmed by our correlational analysis showing a moderate association between CM load and relationship problems. Additionally, in the present study, male partners of women with BPD reported significantly higher prevalence rates of physical neglect and the experience of inconsistency with large effect sizes. They also experienced higher rates of sexual and emotional abuse during childhood on a trend level than their healthy male counterparts. To our knowledge, this is the first study to investigate CM exposure in men having a relationship with women diagnosed with BPD. Therefore, the impact of increased experiences of childhood sexual abuse in male BPD patients on relationship dysfunctions in couples where the female partner suffers from BPD remains unclear. However, previous research on traumatized individuals experiencing childhood sexual abuse has revealed tremendous impairments in the ability to engage in romantic relationships later in life [82,83,84,85]. Nevertheless, due to the relatively low sample size of the present study, as well as the lack of a diagnostic interview for the male partners of the investigated sample, it would be too speculative to draw definite conclusions based on this initial data. Future research should therefore include a larger sample size and a diagnostic assessment of the BPD partners to evaluate the presence of mental disorders, such as post-traumatic stress disorder (PTSD), as well as the presence of CM exposure, which could contribute to the development and maintenance of relationship dysfunction in couples in which one partner suffers from BPD. 

Another important aspect associated with relationship functioning in general are the personality characteristics of each individual within a dyad. In the present study, we found large effects for increased levels of neuroticism, as well as decreased levels of extraversion, conscientiousness, and agreeableness in women with BPD compared to women in the control group. These findings follow previous research assessing BPD specific diagnostic criteria from a Five-Factor Model (FFM) perspective [86,87,88,89]. Specifically, a meta-analysis by Samuel and Widiger [87] revealed a positive correlation between the severity of BPD-related symptoms and angry hostility, self-consciousness, impulsiveness, vulnerability, anxiousness, and depressiveness, all of which are facets of both the personality trait neuroticism and the diagnostic criteria of BPD. Additionally, the same authors [87] showed a negative association between BPD and aspects of positive emotions and warmth within the personality trait extraversion, as well as a negative relationship between BPD and the personality trait conscientiousness, specifically drawing on the aspects of self-discipline, deliberation, dutifulness, and competence. In the male partners of women with BPD, the present study revealed large effects for higher levels of neuroticism, as well as higher levels of openness to new experiences in BPD partners compared to partners of the control group. This finding is interesting and warrants further research into the specifics of the investigated differences, as it might be possible that the neurotic characteristics of the BPD partner contribute to the maintenance of BPD symptoms, as well as relationship disruptions. This notion aligns with the general finding in our study revealing a strong and positive correlation between neuroticism and relationship problems across all included participants. Additionally, it is curious that BPD partners displayed elevated scores on the scale openness compared to partners within the control group, with high scores indicating an increased openness to new experiences, as well as a likelihood to experience positive and negative emotions more intensely. This specific trait might allow BPD partners to be more open to a partner with BPD symptomatology, while at the same time, the intense experience of positive and negative emotions in BPD partners might increase emotion dysregulation in the partner diagnosed with BPD. This interplay of high expressed and felt emotions could ultimately result in dysfunctional interpersonal coping mechanisms while overcoming intensified emotions. Due to the lack of previous research on BPD partners´ personality traits, future investigations could focus on examining the psychopathology as well as the personality characteristics of BPD partners while investigating the relationship between partner psychopathology and relationship disruptions in BPD couples. 

Looking at relationship parameters within our investigated sample, our results were confirmed for the comparison between females with and without BPD: Females diagnosed with BPD were less satisfied within their romantic relationship and displayed fewer positive and more negative dyadic coping styles. Additionally, females with BPD reported more relationship-related problems than their healthy counterparts. This finding aligns with previous research showing lower relationship satisfaction scores and more dysfunctional communication patterns in women with BPD compared to healthy females [37]. However, male partners did not show any differences regarding relationship-related parameters. Additionally, there were no differences regarding relationship satisfaction, dyadic coping, or relationship-related problems within the BPD couple. In turn, healthy females differed significantly from their male partners as they reported fewer relationship-related problems, as well as fewer negative coping styles, while at the same time, were more satisfied with their relationship. Looking at the presented results, the overall findings show that BPD couples are less satisfied with their relationship in comparison to healthy females; however, healthy females were overall more satisfied with their relationship than their male partners. One reason for the discrepancy in relationship satisfaction in healthy women might be the moderating effect of attachment styles. For example, past research showed that attachment avoidance was negatively correlated with relationship satisfaction [90]. Since healthy women within this sample were low on attachment avoidance, and BPD females high, the result could possibly be due to differences in attachment styles. 

Contradicting our initial hypothesis, neither females diagnosed with BPD nor their romantic partners displayed lower cortisol levels than the healthy CG. This finding is interesting as previous research has shown blunted baseline and cortisol stress responses in patients diagnosed with BPD in general [47]. However, previous research also demonstrated the counteracting influences of certain parameters, such as hormonal contraception intake, excessive exercise [91], or a comorbid diagnosis of post-traumatic stress disorder (PTSD) [92,93] on cortisol levels. Specifically, the psychiatric diagnosis of PTSD or depression could be of relevance in the present study since most of the females in the BPD group reported CM experiences as well as depressive symptoms, which could have affected cortisol levels beyond BPD symptomatology. Since the present study has neglected to investigate moderator analysis due to low sample sizes, future studies conducted on larger samples could focus on taking endocrinologically relevant factors into account. 

Interestingly, females with BPD did not differ from females within the control group regarding their baseline testosterone levels. This non-finding could be due to the study design as past research has shown that high testosterone levels were solely associated with externalizing behavior if cortisol levels were low and levels of emotional instability and disagreeableness were high [94]. Future research could therefore separate BPD females according to the proclaimed variables, distinguishing the sample according to high and low levels of each trait. Looking at male BPD partners, our study revealed that BPD partners displayed significantly lower baseline testosterone levels than HC partners. This finding is interesting as it relates to the notion of Porcelli and colleagues [38] who stated that the prevalence of insecure attachment styles in BPD partners reflects their desire to forego severe reactions from the partner diagnosed with BPD. Since high concentrations of exogenous testosterone compromise [95] the capability to accurately detect the emotions and thought process of the colloquist, low testosterone levels in BPD partners appear to be necessary to be able to quickly adapt to BPD related mood swings, and thus prevent severe interpersonal conflicts. 

## 5. Conclusions

To our knowledge, this is the first study to investigate the hormonal as well as personality-related underpinnings of both partners within a romantic dyad where the female partner is diagnosed with BPD. Therefore, it extends previous research evaluating important underlying parameters of couple dysfunction in BPD. We were able to demonstrate that females with BPD display high levels of insecure attachment styles and neurotic personality characteristics, which could be underlying factors contributing to relationship-relevant BPD symptomatology, such as frequent interpersonal conflict, or high expressed emotions. Additionally, we were able to identify CM exposure, as well as insecure attachment styles and neurotic personality characteristics in male partners of patients with BPD, which all together could contribute to symptom maintenance in BPD as well as relationship disruptions. Moreover, BPD partners showed comparably lower testosterone levels than partners in the control group, possibly indicating their desire to avoid relationship related conflicts, as well as symptom acerbation within their partner. Based on the presented findings on BPD partner characteristics, it might be important to incorporate a couple-based approach within BPD psychotherapy to be able to address dysfunctional relationship patterns, which might not solely be caused by the partner diagnosed with BPD. However, due to the relatively low sample sizes, the results should be replicated in larger cohorts and therefore interpreted with caution. Despite these limitations, a total of 52 participants who were recruited within a time-frame of 2 years took part in this study. All participants were selected based on extensive inclusion and exclusion criteria, which was increasingly difficult due to the severity of BPD symptoms within the investigated sample. Nevertheless, future studies could examine these preliminary findings by recruiting larger sample sizes together with including couples where the male and not the female partner is diagnosed with BPD since there are indications that hormonal and interpersonal parameters are sex-specific. 

## Figures and Tables

**Table 1:** Sample characteristics for female participants.

	BPD (n = 14)	CG (n = 12)	Statistics	*p*
**Age in years** (M/SD)	25.90 (6.84)	22.36 (1.95)	t(10.05) = −1.59	0.142
**BMI in kg/m^2^** (M/SD)	27.08 (7.92)	22.82 (3.54)	t(11.59) = −1.59	0.138
**Relationship duration** ^1^	18.00 (15.02)	36.64 (20.58)	t(26) = 2.74	≤0.01
**Level of education**	0/5/5/4	0/1/2/9	X^2^(2) = 5.06	0.08
(no degree/main/middle/high school *)				
**Oral contraception** (no/yes)	7/7	4/8	X^2^(1) = 1.35	0.246
**Current smoking** (no/yes)	8/6	10/2	X^2^(1) = 1.82	0.178
**BDI-II** (M/SD)	32.64 (12.74)	1.64 (2.59)	t(26) = −9.26	<0.001
**GHQ-12** (M/SD)	20.79 (7.44)	7.57 (3.11)	t(26) = −6.36	<0.001
**SCID-II Personality Disorder** (M/SD) ^2^	6.36 (1.82)	0.64 (1.15)	t(26) = −9.92	<0.001
Anxious-Avoidant PD	86%	7%		
Dependent PD	57%	7%		
OCDP	50%	21%		
Negativistic PD	71%	7%		
Depressive PD	86%	0%		
Paranoid PD	50%	0%		
Schizotypical PD	21%	7%		
Schizoid PD	43%	0%		
Histrionic PD	21%	0%		
Narcissistic PD	0%	0%		
Borderline PD	100%	0%		
Antisocial PD	50%	14%		

*Note*: M = Mean; SD = Standard Deviation; BPD = Borderline personality disorder; CG = Control group; BMI = Body mass index, BDI = Beck depression inventory; GHQ = General health questionnaire; PD = Personality Disorder; * = High school in Germany, so-called “Gymnasium”; ^1^ Relationship duration average in months; ^2^ = Multiple diagnoses in each participant were possible.

**Table 2:** Sample characteristics for male participants.

	Partner BPD	Partner CG	Statistics	*p*
	(n = 12)	(n = 12)		
**Age in years** (M/SD)	28.88 (10.41)	26.92 (6.79)	t(18) = −0.51	0.12
**BMI in kg/m^2^** (M/SD)	27.19 (5.97)	26.05 (6.02)	t(18) = −0.42	0.68
**Level of school education**	1/2/3/6	0/0/6/6	X^2^(3) = 4.00	0.26
(no degree/main/middle/high school *)				
**Current smoking** (no/yes)	7/5	9/3	X^2^(1) = 0.750	0.39
**BDI-II** (M/SD)	4.92 (3.32)	10.82 (11.86)	t(21) = −1.73	0.09
**GHQ-12** (M/SD)	12.09 (9.24)	9.25 (3.41)	t(21) = −1.04	0.31
**SCID-II Personality Disorder** (M/SD) ^1^	2.00 (1.67)	1.58 (2.15)	t(21) = −0.52	0.61
Anxious-Avoidant PD	17%	8%		
Dependent PD	8%	0%		
OCDP	42%	42%		
Negativistic PD	17%	8%		
Depressive PD	8%	8%		
Paranoid PD	25%	8%		
Schizotypical PD	8%	8%		
Schizoid PD	8%	8%		
Histrionic PD	8%	17%		
Narcissistic PD	8%	0%		
Borderline PD	8%	0%		
Antisocial PD	50%	50%		

*Note*: M = Mean; SD = Standard Deviation; BPD = Borderline personality disorder; CG = Control group; BMI = Body mass index, BDI = Beck depression inventory; GHQ = General health questionnaire; PD = Personality Disorder; * = High school in Germany, so-called “Gymnasium”; ^1^ = Multiple diagnoses in each participant were possible.

**Table 3:** Attachment, Trauma, Personality: Marginal Means, Significance, and Effect Size.

	Females				Males			
	BPD	CG	F(df)	*p*	Partner BPD	Partner CG	F(df)	*p*
	(M/SE)	(M/SE)	ES (95% CI)		(M/SE)	(M/SE)	ES (95% CI)	
**Attachment Styles**								
**Anxiety**	82.86 (3.94)	35.57 (3.94)	72.14 (26.1)	**<0.001**	53.36 (5.27)	38.83 (5.04)	3.97 (21,1)	**≤0.05**
			**3.1 (1.97–4.21)**				**0.8 (0.05–1.65)**	
**Avoidance**	47.57 (3.89)	27.14 (3.94)	13.81 (26.1)	**<0.001**	44.55 (3.77)	32.25 (3.61)	5.54 (21,1)	**<0.05**
			**1.4 (0.53–2.18)**				**0.9 (0.07–1.80)**	
**Childhood Maltreatment**								
**Overall CM Load**	104.63 (2.60)	86.43 (2.60)	24.46 (26.1)	**<0.001**	96.36 (2.10)	88.58 (2.01)	7.15 (21,1)	**≤0.01**
			**1.8 (0.91–2.69)**				**1.0 (0.17–1.93)**	
Emotional abuse	21.64 (0.73)	15.57 (0.73)	34.63 (26.1)	**<0.001**	19.00 (0.76)	17.17 (0.73)	3.05 (21,1)	0.09
			**2.1 (1.20–3.09)**				0.7 (0.16–1.53)	
Physical abuse	18.43 (0.69)	15.14 (0.69)	11.43 (26.1)	**<0.01**	16.55 (0.50)	15.58 (0.48)	1.92 (21,1)	0.18
			**1.2 (0.42–20.4)**				0.5 (0.29–1.38)	
Sexual abuse	18.79 (0.94)	14.79 (0.94)	9.15 (26.1)	**<0.01**	16.46 (0.87)	14.17 (0.84)	3.59 (21,1)	0.07
			**1.1 (0.30–1.90)**				0.8 (0.09–1.61)	
Emotional neglect	8.21 (0.58)	5.07 (0.58)	14.53 (26.1)	**<0.001**	6.09 (0.44)	5.58 (0.43)	0.68 (21,1)	0.42
			**1.4 (0.56–2.22)**				0.3 (−0.5–1.15)	
Physical neglect	12.71 (0.47)	11.50 (0.47)	3.35 (26.1)	0.079	13.00 (0.43)	11.50 (0.41)	6.31 (21,1)	**<0.05**
			0.7 (0.10–1.43)				**1.0 (0.12–1.86)**	
Experience of inconsistency	13.21 (0.41)	9.71 (0.41)	35.72 (26,1)	**<0.001**	12.00 (0.54)	10.17 (0.52)	6.02 (21,1)	**<0.05**
			**2.2 (1.23–3.12)**				**1.0 (0.10–1.83)**	
**Personality Traits**								
**Neuroticism**	3.14 (0.18)	1.40 (0.14)	60.63 (20.1)	**<0.001**	1.68 (0.19)	1.03 (0.18)	6.02 (21,1)	**<0.05**
			**3.4 (2.01–4.72)**				**1.0 (0.10–1.84)**	
**Extraversion**	1.47 (0.13)	2.70 (0.10)	55.87 (20.1)	**<0.001**	2.06 (0.17)	2.49 (0.16)	3.31 (21,1)	0.08
			**2.9 (1.62–4.10)**				0.7 (0.12–1.57)	
**Openness**	2.67 (0.18)	2.29 (0.14)	2.76 (20.1)	0.113	3.02 (0.14)	2.27 (0.14)	14.51 (21,1)	**<0.001**
			0.7 (0.19–1.60)				**1.5 (0.57–2.44)**	
**Conscientiousness**	2.37 (0.20)	2.95 (0.15)	5.81 (20.1)	**<0.05**	2.36 (0.17)	2.76 (0.16)	2.93 (21,1)	0.1
			**1.0 (0.07–1.92)**				0.7 (0.16–1.53)	
**Agreeableness**	2.43 (0.20)	2.95 (0.15)	4.53 (20.1)	**<0.05**	2.42 (0.15)	2.53 (0.15)	0.27 (21,1)	0.61
			**0.9 (0.06–1.76)**				0.2 (−0.63–1.01)	

*Note*: BPD = Borderline personality disorder; CG = Control group; M = Mean; SE = Standard Error; ES = Effect size based on Cohen´s d; CI = Confidence Interval; CM = Childhood Maltreatment.

**Table 4:** Relationship Parameters between BPD and healthy controls by sex.

	Females				Males			
	BPD (M/SE)	CG (M/SE)	F(df) ES (95% CI)	*p*	Partner BPD (M/SE)	Partner CG (M/SE)	F(df) ES (95% CI)	*p*
**Partnership Questionnaire**								
**PFB Sum**	66.00 (3.20)	76.57 (3.20)	5.45 (26,1) **0.9 (0.07–1.63)**	**<0.05**	66.64 (3.25)	68.00 (3.11)	0.09 (21,1) 0.1 (−0.7–0.94)	0.76
**Questionnaire for Assessing Dyadic Coping**								
**QADC Sum**	89.00 (6.91)	106.64 (4.13)	4.81 (17,1) **1.0 (0.08–2.07)**	**<0.05**	95.75 (4.35)	93.75 (3.32)	0.07 (17,1) 0.1 (−1.05–0.82)	0.80
Positive Coping	106.80 (6.72)	121.00 (4.02)	3.29 (17,1) 0.9 (0.19–1.94)	0.087	116.43 (3.76)	112.58 (2.87)	0.66 (17,1) 0.4 (−0.59–1.29)	0.43
Negative Coping	17.80 (1.41)	14.36 (0.85)	4.37 (17,1) **0.9 (0.15–1.98)**	**≤0.05**	21.29 (1.84)	18.83 (1.40)	1.13 (17,1) 0.5 (−0.43–1.46)	0.30
**Problem List**								
**PL Sum**	13.83 (1.58)	6.50 (1.58)	10.81 (22,1) **1.3 (0.45–2.23)**	**<0.01**	14.00 (2.27)	10.91 (2.18)	0.96 (21,1) 0.4 (−0.43–1.12)	0.34

*Note*: BPD = Borderline personality disorder; CG = Control group; M = Mean; SE = Standard Error; ES = Effect size based on Cohen´s d; CI = Confidence Interval; Sum = Sum score; PFB = Partnership Questionnaire; QADC = Questionnaire for Addressing Dyadic Coping; PL = Problem List.

**Table 5:** Correlational Analysis between Relationship Problems and Predispositioning Factors, across the Entire Sample.

	ECR		CTQ							NEO-FFI
	Anxiety	Avoidance	EA	PA	SA	EN	PN	EI	Overall	Neuroticism
PL	0.465 ***	0.525 ***	0.405 **	0.338 *	0.069	0.073	0.256	0.327 *	0.324 *	0.482 ***

*Note*: PL = Problem List; ECR = Experience of Close Relationship Scale; Anxiety = Attachment Anxiety; Avoidance = Attachment Avoidance; CTQ = Childhood Trauma Questionnaire; EA = Emotional Abuse; PA = Physical Abuse; SA = Sexual Abuse; EN = Emotional Neglect; PN = Physical Neglect; EI = Experience of Inconsistency; Overall = CTQ Overall Score; * = significant on the 0.05 level; ** = significant on the 0.01 level; *** = significant on the 0.001 level.

## Data Availability

The datasets generated during and analyzed during the current study are available from the corresponding author on reasonable request.

## Supplementary Materials

The following supporting information can be downloaded at: https://www.mdpi.com/article/10.3390/bs13030253/s1, Figure S1: Attachment Parameters: Marginal Means & Significance; Figure S2: Overall CM Load, Sexual Abuse, & Experience of Inconsistency: Marginal Means & Significance; Figure S3: Physical Abuse and Physical Neglect: Marginal Means & Significance; Figure S4: Emotional Abuse and Emotional Neglect: Marginal Means & Significance; Figure S5: Neuroticism & Extraversion: Marginal Means & Significance; Figure S6: Openness, Conscientiousness, and Agreeableness: Marginal Means & Significance.
