# Supplementary material for: Relationship Dysfunction in Couples When One Partner Is Diagnosed with Borderline Personality Disorder: Findings from a Pilot Study
Source: Behav Sci (Basel). 2023 Mar 13;13(3):253. doi: 10.3390/bs13030253 (PMC10045094; doi:10.3390/bs13030253)
Supplement: Supplementary file 1 [file behavsci-13-00253-s001.zip › behavsci-2227386-supplementary.pdf]

Figure S1. Attachment Parameters: Marginal Means & Significance.

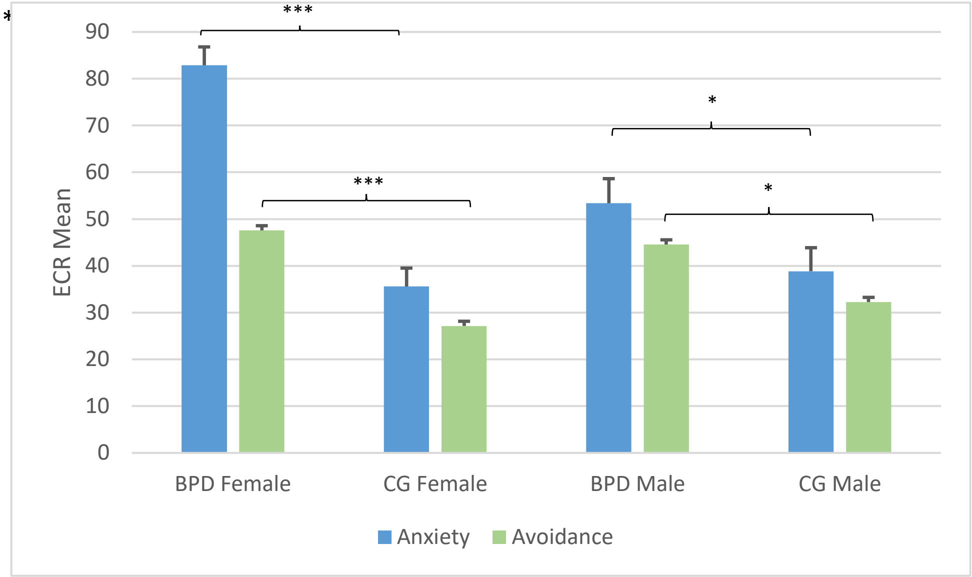

Note: ECR = Experiences in Close Relationships; BPD = Borderline Personality Disorder; CG = Control Group. * = p < 0 .05; *** = p < 0.001.

Figure S2. Overall CM Load, Sexual Abuse, & Experience of Inconsistency: Marginal Means & Significance.

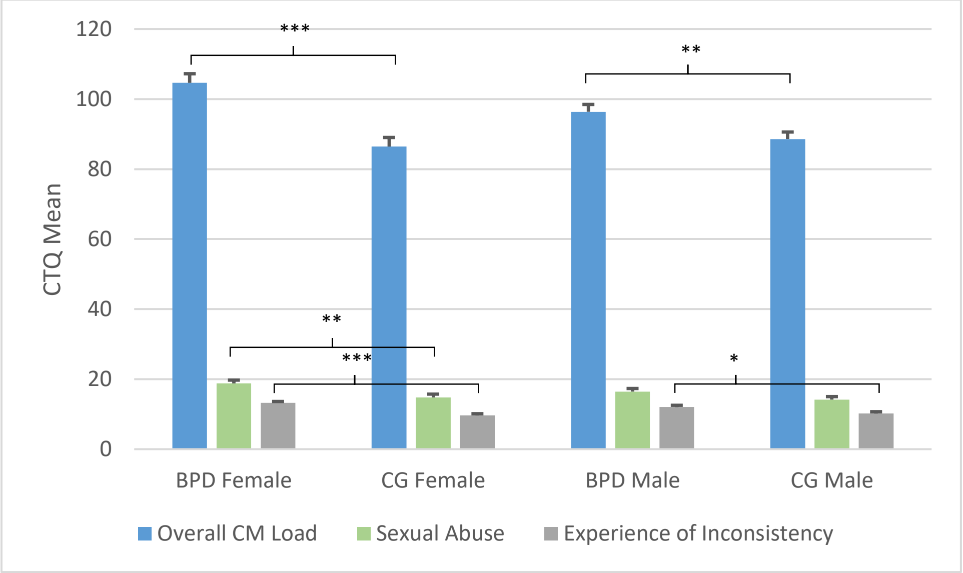

Note: CTQ = Childhood Trauma Questionnaire; BPD = Borderline Personality Disorder; CG = Control Group; Overall CM Load = Overall Childhood Maltreatment Load. * = p < 0.05; ** = p < 0.01; *** = p < 0.001.

Figure S3. Physical Abuse and Physical Neglect: Marginal Means & Significance.

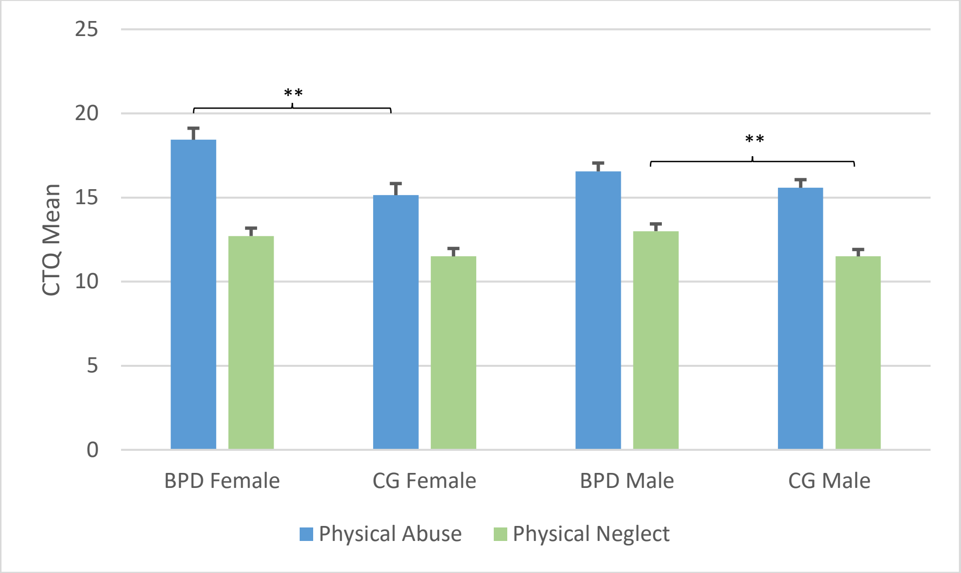

Note: CTQ = Childhood Maltreatment Questionnaire; BPD = Borderline Personality Disorder; CG = Control Group.** = p < 0.01.

Figure S4. Emotional Abuse and Emotional Neglect: Marginal Means & Significance.

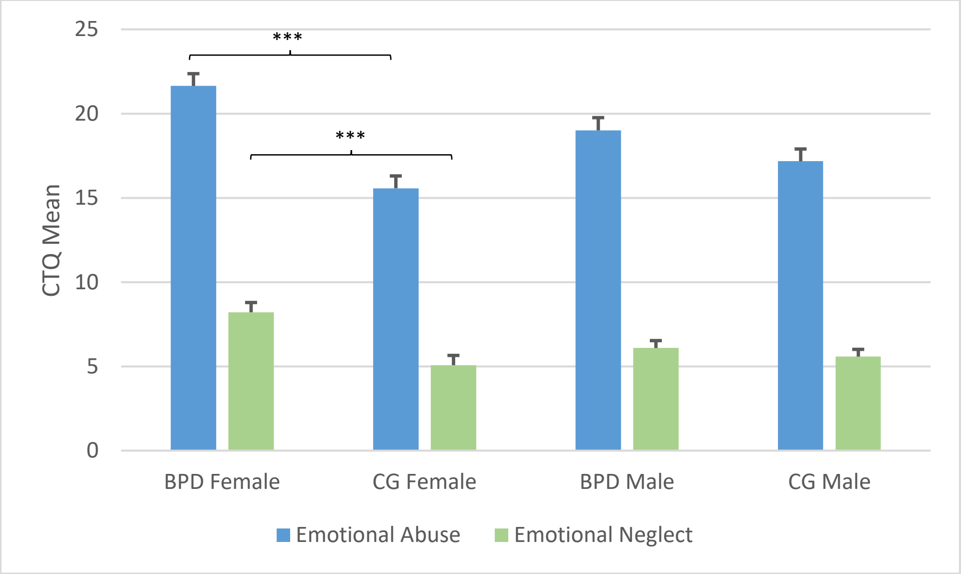

Note: CTQ = Childhood Maltreatment Questionnaire; BPD = Borderline Personality Disorder; CG = Control Group.*** = p < 0.001.

Figure S5. Neuroticism & Extraversion: Marginal Means & Significance.

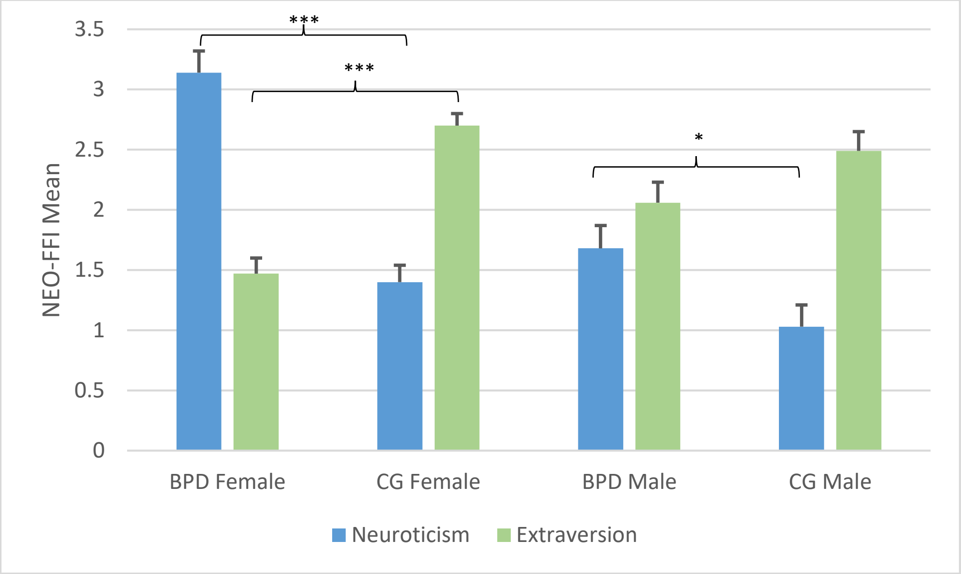

Note: NEO-FFI = NEO Five-Factor-Inventory; BPD = Borderline Personality Disorder; CG = Control Group.* = $p < 0.05$; *** = $p < 0.001$.

Figure S6. Openness, Conscientiousness, and Agreeableness: Marginal Means & Significance.

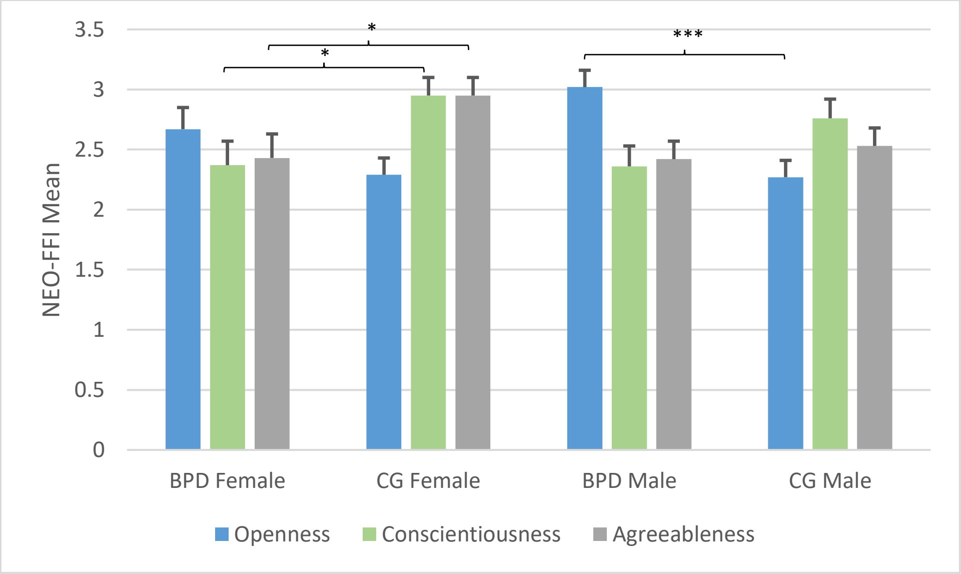

Note: NEO-FFI = NEO Five-Factor-Inventory; BPD = Borderline Personality Disorder; CG = Control Group.* = $p < 0.05$; *** = $p < 0.001$.
